# How biotic and abiotic effects colour flowers in a land Down Under

**DOI:** 10.1111/nph.16856

**Published:** 2020-08-29

**Authors:** Anne C. M. Verloop, Adrian G. Dyer, Casper J. van der Kooi

**Affiliations:** ^1^ Groningen Institute for Evolutionary Life Sciences University of Groningen Groningen 9747AG the Netherlands; ^2^ Bio‐inspired Digital Sensing (BIDS) Lab School of Media and Communication RMIT University Melbourne Vic 3000 Australia

**Keywords:** biotic and abiotic factors, colour vision, evolution, floral pigments, flower colour variation, pollination, reflection, temperature

## Abstract

This article is a Commentary on Dalrymple *et al*. (2020), **228**: 1972–1985.

Angiosperms display an astonishing diversity of flower colours. Floral coloration primarily evolved because visual signals enable attraction of animal pollinators. Indeed, flower coloration evolved to be conspicuous in the eyes of pollinators (Chittka & Menzel, 1992; Lunau *et al*., 2011; Dyer *et al*., 2012; Shrestha *et al*., 2013; van der Kooi *et al*., 2019a). In addition to pollinators as agents of selection, abiotic factors may also shape floral coloration. In this issue of *New Phytologist*, Dalrymple *et al.* (2020; pp. 1972–1985) examine how common biotic and/or abiotic factors shape floral coloration in Australia.

‘An important finding of their study is that flowers tend to be more colourful in stressful growing conditions.’

Dalrymple *et al*. studied flower colours of 339 species from 74 families, covering tropical, temperate, arid, montane and coastal environments in Australia. To test 10 predictions for how biotic and abiotic factors shape flower coloration, they included 11 environmental variables, such as plant and animal diversity, geographic location and various climatic factors. Biotic factors were found to be most important in explaining flower colour variation, with insect community diversity being the strongest explanatory variable. However, there is more to understanding flower colour variation, and, surprisingly, several conventional assumptions were not upheld by their findings from this large continental island.

A major aspect of floral visual signals is colour contrast, for example between the flower and background, or within‐flowers in the case of colour patterns (Fig. 1). Colour contrast is determined both by the flower's hue (which is commonly called ‘colour’) and saturation (which is the ‘purity’ of a colour) (van der Kooi *et al*., 2019a). In their model, Dalrymple *et al*. included pollinator diversity, primary production and length of the growing season, and solar radiation and precipitation as important proxies for plant stress. An important finding of their study is that flowers tend to be more colourful in stressful growing conditions. As an example, they found that in northern Australia, where solar exposure is *c*. 25% higher, colour contrast of flowers is *c*. 2.5% higher than 2500 km away in the southern island of Tasmania. Similarly, inland species of the continent receive about a third of the precipitation compared to coastal species, but exhibit *c*. 4% more colour contrast.

**Fig. 1:**
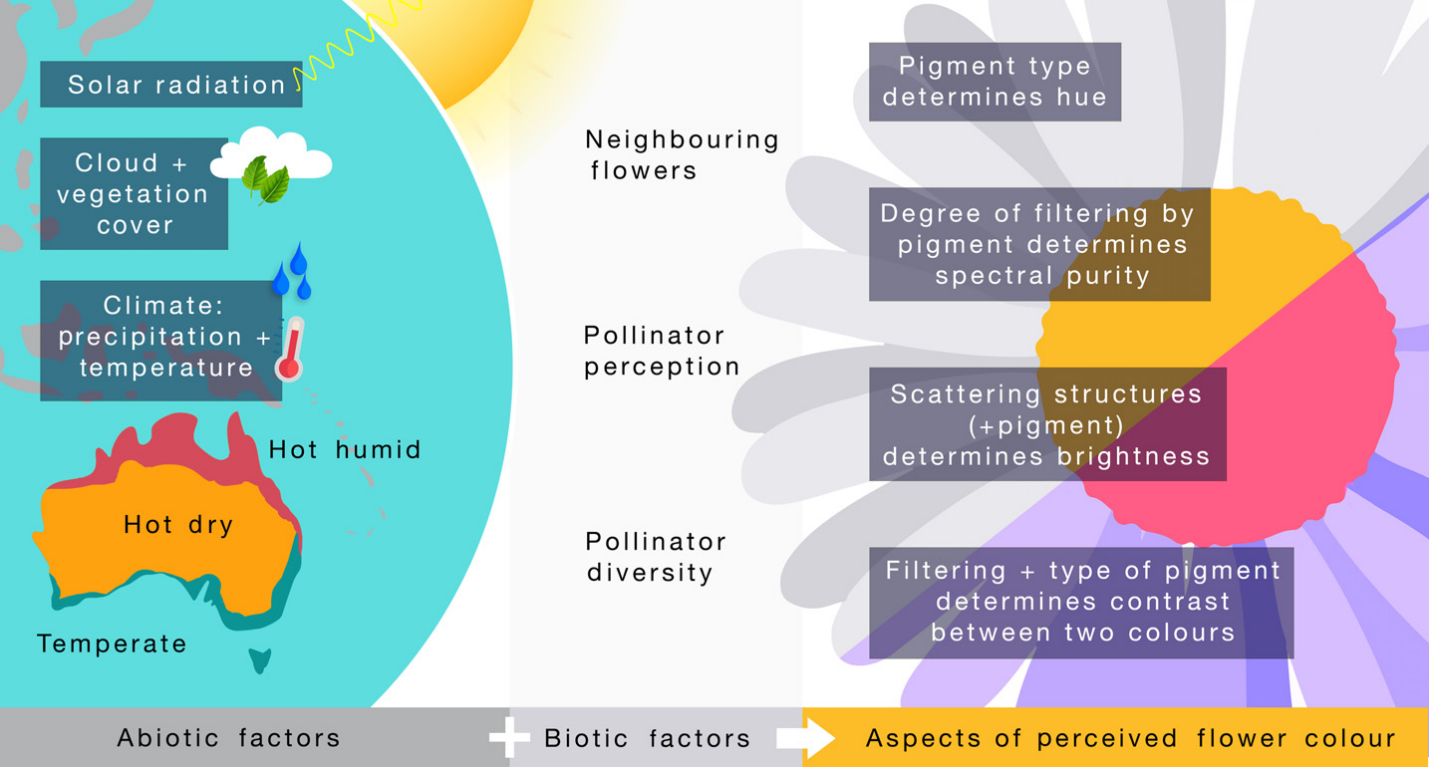
Abiotic and biotic effects as evolutionary agents of selection on flower colour. The Australian continent exhibits marked variation in solar radiation, precipitation, temperature, and plant and pollinator diversity, which provides a spatially varying selection landscape on the optical properties of flowers.

How then do abiotic stressors impact flower coloration? Production of anthocyanins and flavonols can increase as a response to solar radiation (Falcone Ferreyra *et al*., 2012), which may lead to more saturated flowers. Pigments can have specific functions in protecting flowers from abiotic stressors. For example, increasing the amount of ultraviolet absorbing pigments can reduce the amount of (harmful) ultraviolet light that is reflected to the pollen (Koski & Ashman, 2015). Alternatively, though not mutually exclusive, is that changes in flower pigmentation are a side effect of the stress response on the plant. Warren & Mackenzie (2001) found that plants with higher anthocyanin levels throughout the entire plant have higher fitness under drought stress, whereas plants with low anthocyanin levels performed better in the well watered conditions. Nonetheless, the amount of pigment cannot fully explain differences in colour contrast as found by Dalrymple *et al*., as colour contrast is also mediated by the type of pigment and where it is located inside the flower (van der Kooi *et al*., 2019a). Currently however, we know little about how the synthesis of other floral pigments, such as carotenoids and betalaines, relate to abiotic stress.

Australia is well known for its temperature variation and extremes. An intuitive role of colour with regard to temperature is that flowers with darker hues will convert more radiation to heat than flowers with light hues, although this may be more clearly linked to solar radiance than ambient temperature. Further, (chemical) developmental processes of plants as well as pollinator foraging activities are temperature dependent (van der Kooi *et al*., 2019b). Perhaps surprising, therefore, is that in their large dataset Dalrymple *et al*. found no association between ambient temperature and colour contrast. The absence of an effect may be because colour contrast does not directly scale with pigmentation in a simple way. The optical properties of pigments that produce salient colours like yellow that contrast well with a (green) background may only require a bit of pigmentation, and increasing pigmentation to increase photon catch (and so passive heating of the flower) may actually decrease visual colour contrast to the background (van der Kooi *et al*., 2019a). It is further likely that plant physiological processes related to thermal tolerance will play a key role here also.

Many flowers display colour patterns, which can function as a guide for pollinators to find the pollen and nectar. Dalrymple *et al*. tested whether presence and type of colours in such patterns are correlated with plant and/or pollinator species richness. The theory of character displacement dictates that with increasing plant diversity floral signals should diverge, so to reduce interspecific pollen transfer and competition for animal pollination service. In contrast with this hypothesis, Dalrymple *et al*. found that within‐flower colour patterns converged with increasing plant species diversity. With increasing (pollinating) bird species richness, colour patterns also become more similar in hue; colour patches shift to orange–red wavelength ranges instead of the more contrasting orange–purple. There was no clear effect of insect community diversity on within‐flower colour contrast.

At least three nonmutually exclusive factors can explain the observed convergence in flower colour patterns. First, floral signal convergence could be explained by increased sharing of pollinators in diverse communities (i.e. more generalized pollination), because plants with more generalized pollination systems are more successful in such environments than specialists. Convergence of floral (visual) signals will then be particularly useful when co‐flowering plants are neighbours and – from a distance – constitute a large floral display. Second, other pollinator‐attracting signals, such as scent, size or shape, may become increasingly important in diverse communities. Third, pollinators may use fine colour differences more in highly diverse communities, meaning that (small) differences in colour are more important in species‐rich than species‐poor communities. Testing the relative importance of these different predictions requires detailed characterization of pollinators in the focal communities, which will be challenging owing to the vast geographic range. In this regard, within Australia, another recent community study revealed flower colour signals tend to converge towards the visually mediated colour preferences of key insect pollinators such as bees or flies (Shrestha *et al*., 2019).

Adding to the complexities of comparing biotic and abiotic influences at a continental scale was Dalrymple *et al*.'s observation of no clear effect of insect community diversity on within‐flower colour contrast. Insect colour vision greatly varies between taxa, and insect foraging can further be guided by colour preferences and achromatic (green) contrast (Fig. 1, van der Kooi *et al*., 2019a). Together, this suggests that particularly for insect‐pollinated flowers, understanding the evolution of flower colours requires incorporating pollinator visual systems. Dalrymple *et al*. did not include different visual systems in their analysis, which is legitimate given the breadth of their research and our current poor knowledge of colour vision in many (Australian) pollinators.

Another factor that determines the visual signal of flowers is the amount of the reflected light, which humans perceive as brightness. The amount of the reflected light depends on both pigments and flower structure. Light is reflected by structures inside the flower (e.g. cell walls, air gaps, vacuoles), and reflected light is modulated by wavelength‐selective absorption by floral pigments (van der Kooi *et al*., 2019a). To humans, for example, white and yellow flowers appear brighter than red flowers. Dalrymple *et al*. found that the amount of light reflected by flowers increases with vegetation cover (quantified using leaf area index). In this regard the findings by Dalrymple *et al*. differ from those from Binkenstein & Schaefer (2015), who found no difference between colours of flowers between forests and open grasslands in Germany. The amount of the reflected light – independent of the colour – is unlikely to be important for many (diurnal) insect pollinators; behavioural tests suggest these insects do not process ‘brightness’ signals (for review, see van der Kooi *et al*., 2019a). Perhaps the increased floral reflectance in Australian forest‐dwelling species is because of structural differences in flower anatomy that are related to mechanical aspects or flower longevity more than the colour signal.

The work by Dalrymple *et al*. made clear that both biotic and abiotic factors in studies on the evolution of flower colour are important, and should be investigated in floras on other continents. The main strength of the paper of Dalrymple *et al*. is their impressive taxonomic and geographic coverage (339 species at several habitats). However, not uncommon in such taxonomically vast studies, is that their breadth comes at the expense of detail. As acknowledged by Dalrymple *et al*., multiple significant results in this study have a low effect size. We also need a more sophisticated map of the ecological network of plant and pollinator species to understand the effects of pollinator diversity and different pollinator species on floral coloration on both local community level and a broader scale. Future research will no doubt illuminate how biotic and abiotic effects contribute to colouring the flora Down Under and in other parts of the world.
